# University Students’ Satisfaction with the Quality of Primary Dental Healthcare Services and Dentists in Croatia: A Cross-Sectional Study

**DOI:** 10.3390/clinpract13010005

**Published:** 2022-12-30

**Authors:** Antonija Tadin, Marijeta Dadic, Lidia Gavic

**Affiliations:** 1 Department of Restorative Dental Medicine and Endodontics, Study of Dental Medicine, School of Medicine, University of Split, 21000 Split, Croatia; 2 Department of Maxillofacial Surgery, Clinical Hospital Centre Split, 21000 Split, Croatia; 3 Private Dental Practice, 20350 Metkovic, Croatia

**Keywords:** dental care, dental service, healthcare quality, patient preference, patient satisfaction

## Abstract

Measuring satisfaction can be an essential method for evaluating and improving healthcare quality. Therefore, this survey aimed to determine university students’ satisfaction with dental care at the primary healthcare level and the factors that influence students’ decisions when choosing a dental provider. A cross-sectional survey was conducted using a self-administered electronic questionnaire that assessed satisfaction with various aspects of dental care (patient–staff interaction, professional and technical competence, and administrative efficiency) on a 5-point Likert scale. A total of 806 students participated in the survey, of whom 56.6% were from a biomedical science background, and 43.4% from other scientific fields. Near-minimal differences were found between respondents studying in biomedical fields and those from other scientific fields, when evaluating satisfaction with dental services. More significant differences were found in the factors influencing their choice of dentist. The dentist’s experience (*p* = 0.031), cost of service (*p* ≤ 0.001), office location (*p* = 0.034), waiting time (*p* = 0.029), qualifications (*p* = 0.033), and gender (*p* = 0.007) were more important for students pursuing one of the non-health-related majors. Overall, respondents were very satisfied with their dentists and the services provided. The highest satisfaction score was found on the “professional and technical competence” and “administrative efficiency” subscales, while the lowest satisfaction score was found on the “patient–staff interaction” subscale.

## 1. Introduction

A high-quality healthcare system needs to adhere to contemporary knowledge in health procedures, ensure the best possible patient outcome, and reduce the risk of undesirable side-effects. It represents one of the fundamental human rights [1]. The perception of the quality of health services depends on the stakeholders’ priorities and attitudes towards the health system in general [2]. Patients and healthcare professionals are equally involved in the quality of health service provision. Accordingly, factors influencing healthcare service quality are divided into extrinsic ones related to the service provider, and intrinsic ones related to patients. Extrinsic factors include communication skills, technical competence, approach and explanation of dental procedures, availability and convenience, organizational skills, and effectiveness and outcome of care. Intrinsic factors include gender, age, education level, socioeconomic status, marital status, general and mental health status, frequency of visits, personality, anxiety, and general life satisfaction. In addition, the patient is affected by convenience factors such as waiting time in the surgery, waiting time for an appointment, opening hours, and location, as well as treatment costs, office equipment, sterility and cleanliness of the dental office, and pain control [3,4,5,6,7,8].

Measuring patient satisfaction has become increasingly important [9,10]. Satisfaction with healthcare provision is the foundation of public and private healthcare institutions worldwide [11]. In order to evaluate the performance of a health system, it is essential to measure service quality. This is a multidimensional concept defined as safe, effective, people-centred, timely, equitable, integrated, and efficient [2,12,13]. In the past, the health professional was the one who would measure the quality of the healthcare provided. Today, as a “consumer” of healthcare, the patient has a certain level of autonomy and the right to participate in clinical decision-making processes that affect quality of life. The patient’s opinion is essential for monitoring the healthcare system, and ultimately for influencing improvements to healthcare policy. 

Most importantly, healthcare can only fully achieve its goals if patients are satisfied with the service provided [14,15]. Patient satisfaction equates to the patient’s assessment of the services provided and the overall healthcare system, and is commonly used as a quality indicator [16]. When the patient is satisfied, loyalty towards the healthcare provider increases.

Loyal patients tend to follow medical advice, continue to receive services according to prescribed treatment plans, maintain relationships with specific healthcare providers, and are willing to make referrals [17,18]. Therefore, measuring patient satisfaction is essential to determine success, develop strength in today’s highly competitive healthcare market, and, most importantly, improve healthcare in general. Assessing satisfaction from the patient’s perspective has become crucial to healthcare managers who deal with policy analysis in order to improve the quality of healthcare, meet patient expectations, reduce costs, formulate strategies for effective management, and monitor the success of health plans [17,19,20]. As in all branches of medicine, feedback on patient satisfaction with dental care is vital for improving dental service delivery [11,21,22,23].

In Croatia, all healthcare activities, including dental healthcare, must be standardized and equally available in all parts of the country. The Agency for Quality and Accreditation in Health and Social Welfare is a governmental body that carries out mandatory monitoring of healthcare quality indicators for healthcare providers in Croatia [24]. However, there is as yet no available information on patient satisfaction with primary healthcare dental services. Therefore, this survey aimed to evaluate students’ satisfaction with dental services provided at the primary healthcare level, and the factors affecting their decisions when choosing a dental provider. Our hypotheses were that when comparing respondents from different fields of scientific study, there would be no difference in satisfaction with various aspects of dental service provided, and there would be no difference in factors considered necessary when choosing a dentist and dental practice.

## 2. Materials and Methods

This cross-sectional questionnaire-based survey was conducted between March 2021 and April 2021 at the Department of Restorative Dental Medicine and Endodontics, Study of Dental Medicine, School of Medicine, University of Split, Croatia. It was conducted in accordance with standard ethical principles, including the Declaration of Helsinki, and was approved by the University Ethics Committee (Class: 003-08/2L-03 10003, Reg. No: 2181-198-03-04-21-0015). The research was carried out in line with the recommendations and applicable regulations and in compliance with the institutional code of ethics and reporting following the instructions given by STROBE (Strengthening the Reporting of Observational Studies in Epidemiology) [25]. The research was not financially supported and was conducted using voluntary response sampling. The participants could withdraw from the study at any time.

### 2.1. Participants and Settings

The survey was based on a self-administered questionnaire delivered through a network survey (*Google Forms*). All students of the University of Split (over 18 years of age, irrespective of gender and length of study) were invited by the President of the Student Association to participate in the survey, which was distributed electronically through the Facebook platform of the University, which has 6579 members. The questionnaire was completed by students from eighteen different fields of study: four from health fields (medicine, dentistry, pharmacy, and health sciences) and fourteen from other academic areas unrelated to healthcare. 

Respondents were given information about the study in Section 1 of the questionnaire. Inclusion criteria implied adult regular students of the University of Split in the academic year 2020–2021 who had undergone at least one dental procedure in the past year. Exclusion criteria were the age of the student and the non-completion of the questionnaire. The questionnaire was completely anonymously, and respondents confirmed their voluntary participation in the study by completing it. 

The required minimum sample size (*n* = 377) was calculated from the total number of students at the University of Split in the academic year 2020–2021 (*n* = 19,500) with a confidence interval of 95% and a response distribution of 50%.

### 2.2. Questionnaire

The self-designed and self-administered questionnaire was based on several studies on the same topic, consisted of four sections, and comprised 50 questions [11,23,26,27,28,29,30,31]. Section 1 included primary sociodemographic data of the respondents (gender, age, study, year of study, employment of a family member in healthcare, and financial assessment of the family). The second part contained 11 questions related to the respondent’s attitude towards the use of dental services, the chosen dentist, and dental practice. The third part contained 20 questions assessing respondents’ satisfaction with dental care (patient–staff interaction, professional and technical competence, and administrative efficiency). To answer the questions, respondents could select a specific response option reflecting their level of agreement (or disagreement) with a statement on a 5-point Likert scale. Section 4 consisted of 14 questions related to factors that may influence the choice of a dentist. A list of choices was provided for each of these categories, and respondents were instructed to rate their importance on a 5-point Likert scale from ‘not at all important’ to ‘absolutely important’. 

Before the Internet survey was distributed to respondents, two dentists (university professors with expertise in research methodology) reviewed the draft survey. In addition, a prior pilot study was conducted with 30 students (15 medical and 15 non-medical) to check reliability, which confirmed the comprehensibility of the questionnaire. These questionnaires were not included in the primary study data. Internal consistency showed a Cronbach’s coefficient alpha of 0.754 for the portion of the questionnaire that assessed satisfaction with various aspects of dental care, and 0.791 for the portion that considered factors of dentist choice.

### 2.3. Statistical Analysis

Data were analysed using the Statistical Package for the Social Sciences, version 26 (SPSS, IBM Corp, Armonk, NY, USA). The Kolmogorov–Smirnov test was used to assess the normality of the distribution of responses. Descriptive analysis was used to calculate the frequency and percentage of categorical data, while quantitative data were reported as mean and standard deviation. The chi-square test was employed to compare categorical variables among respondents according to their study field (medical care or nonmedical care), while the Mann–Whitney test was used to compare satisfaction scores on a 5-point Likert scale. The Spearman correlation coefficient was used to assess the relationship between respondents’ attitudes and their sociodemographic characteristics. The significance level was set at *p* < 0.05.

## 3. Results

The sociodemographic characteristics of the respondents are presented in Table 1. A total of 806 students from the University of Split participated in the research (12.3% response rate). The mean age of the respondents was 23.39 ± 2.24 (minimum 19, maximum 33), and a narrow majority (56.6%) studied biomedical or health-related subjects.

Table 2 shows the attitude of patients towards their dentist and dental practice. Most respondents (*n* = 338, 41.9%) visit a private dental practice under a contract with a public health insurance company. Around a third of them (34.1%) have never changed dentist, while a similar proportion have changed once (32.4%) or more than once (33.5%). More than three quarters of respondents (77.5%) were very or mostly satisfied with their chosen dentist and dental practice, and 67.5% would recommend them to their family, friends, and acquaintances.

Table 3 shows a comparison of Likert scale ratings in five levels of agreement and disagreement (strongly agree, agree, undecided/neutral, disagree, strongly disagree) given by respondents to various statements related to satisfaction with primary dental care based on patient–dental staff interaction, professional and technical competence of dental staff and dental practice, and administrative efficiency parameters. When comparing the responses of students from biomedical studies with those from other scientific studies, all respondents agreed on all statements except on those related to the painfulness of the dental procedure and the price of dental services. Respondents from biomedical degree programs believe that dental procedures are less painful (3.98 ± 0.98 vs. 3.71 ± 1.09, *p* ≤ 0.001), and regard the cost of dental services as more acceptable (2.77 ± 1.20 vs. 3.01 ± 1.32, *p* = 0.007).

Table 4 represents the results of the correlation between respondents’ attitudes towards satisfaction with primary care dental services and their sociodemographic data (gender, age, study, year of study, family member in healthcare, and type of dental healthcare service used). 

Table 5 shows respondents’ attitudes regarding the most important reason for choosing a particular dentist or dental surgery. 

Table 6 represents the results of the correlation between the most important reason for choosing the dentist and the respondent’s sociodemographic data (gender, age, study, year of study and family member in healthcare). 

## 4. Discussion

Providing quality healthcare should be the primary goal of healthcare providers. Patient satisfaction with the services offered is crucial in measuring and improving quality [2,3,4]. This survey examined students’ satisfaction with services in primary dental healthcare. A total of 806 students from all years of study at the University of Split participated in the study. Its specific objectives were to determine satisfaction with dental care, evaluate the factors influencing the choice of dentist, and investigate the differences in satisfaction levels under the influence of sociodemographic characteristics. The first hypothesis of this study was confirmed: satisfaction levels were generally unrelated to the student’s field of study. Comparing the responses of students from biomedical majors with those of students from other majors, no significant difference in responses was found, except for questions about the cost and the pain of the procedure. Students in biomedical majors considered the procedures less painful (*p* ≤ 0.001) and the price more acceptable (*p* = 0.007). However, the second hypothesis of this study was rejected: there was indeed a correlation between the student’s field of study and factors influencing their choice of dentist. Dentist experience was found to be more critical for students of other science majors than for biomedical science students (*p* = 0.031), as were the price of service (*p* ≤ 0.001), location of practice (*p* = 0.034), waiting time (*p* = 0.029), dentist qualifications (*p* = 0.033), and dentist gender (*p* = 0.007).

In this study, 77.5% of respondents indicated that they were very satisfied or mostly satisfied with their dental service. These results are consistent with a 2015 study conducted in Iran, in which 77% of respondents were satisfied with dental services and baseline condition improvement, while 12% were dissatisfied [32]. A similar level of satisfaction with dental care, 79.5%, was found in a study conducted in Saudi Arabia [27]. In the United Kingdom, up to 89% of respondents were satisfied with the quality of dental services [33]. In a study by Killingerbeng et al. [34] evaluating dental care in Germany, general satisfaction was high. Nevertheless, patients were critical of some aspects of dental care, such as waiting times and treatment costs, which is consistent with the results of our study.

This survey assessed patient–staff interaction, technical and professional competencies, and administrative efficiency. A study in Indonesia used a similar method of determining patient satisfaction [35]. The dimensions of the Indonesian survey were relationships and communication, dental care, information and support, office organization, and physical structure. Respondents were least satisfied with the organization of the dental office due to the long waiting times for an appointment and with the dimension of dental care due to insufficient professional competence. In contrast, the results of our study showed a high level of satisfaction with professional competence and administrative efficiency [35]. A similar study was conducted on a high school population in Malaysia. The Malaysian study aimed to assess satisfaction with dental care provided in schools by mobile dental units, examining four dimensions: patient–staff interaction, technical competence, administrative efficiency, and clinic organization. Of the total respondents in that survey, 62.0% were satisfied with the services provided, somewhat lower than in our study (77.5%). The main reason for dissatisfaction in Malaysia was that the dental staff did not explain the procedure before the treatment (15.4%) [29]. However, the results of our survey show that most responses were positive, i.e., the dentist explained the procedure before treatment (68.6%). In a previous survey conducted in Zagreb, Croatia, 71.35% of respondents stated that they were quite satisfied with the information about the treatment procedure [36]. Of the total number of respondents in the study in Malaysia, 46.0% were dissatisfied with the personality and facial expression of the dental staff [29]. In our survey, 75.6% of the respondents reported that the facial expression of the dentist was friendly and smiling; similar results were obtained in the study in Saudi Arabia (77.8%) [27].

The main objective of a study conducted in Izmir, Turkey, was to investigate the factors of satisfaction and dissatisfaction with dental services. Regardless of sociodemographic differences, interaction was prioritized in order to provide a satisfactory dental service [37]. In our study, patient–staff interaction was the most critical dimension in assessing satisfaction. In the previously-mentioned survey conducted in Zagreb, 78.89% of respondents indicated that they were quite satisfied with their communication with their chosen dentist [36]. A positive patient–dentist relationship leads to a higher satisfaction rate, creating greater engagement and interest in dental treatment and ultimately better outcomes [38]. Rankin and Harris [39] confirm the importance of interaction and putting the patient’s needs first, which leads to a positive experience with the dentist. According to Anderson [7], verbal and nonverbal communication is essential for customer satisfaction because it reduces patient anxiety. On the other hand, Goedhart et al. [40] argue that the technical aspects of treatment take precedence over communication skills.

Pain control has a significant impact on the level of satisfaction. Of the total number of respondents in our study, 67.6% said that the procedures were not painful. Similar results were obtained in the study in Saudi Arabia (68.5%) [27]. In a Norwegian study, the presence of pain during a dental procedure was shown to lead to the postponement of future dental visits [41].

Our study showed a weak relationship between demographic characteristics (gender, age, year of study, family member in health care, and type of dental service) and satisfaction with the dental service provided. Gender has a weak negative correlation with certain factors. For example, tidiness and cleanliness of the office and the dentist (*p* ≤ 0.001), trust and compassion of the dentist (*p* ≤ 0.001), good communication skills (*p*≤ 0.001), and accessibility and friendly behaviour (*p* ≤ 0.001) are less important for men (*p* ≤ 0.001). A study conducted at the University of the West Indies investigated the relationship between patient satisfaction and sociodemographic factors (age, gender, ethnicity, education), frequency of dental visits, and self-assessment of oral health. The results showed no statistically significant relationship between satisfaction and sociodemographic factors, except for self-assessment of oral health. The better the oral health self-assessment, the higher the satisfaction [26]. Arnbjerg et al. [42] studied satisfaction with dental services in the Swedish population and concluded that the age and gender do not influence satisfaction. In contrast, some studies show that women are more satisfied with dental services [43]. A study in Kuwait found that younger people are more satisfied, which is associated with better dental status and less need for extensive procedures [21].

Our study has some limitations. It was conducted using an online questionnaire, so the reliability of the data may be a limiting factor. In anonymous self-reporting studies, the problem of self-assessment bias is ubiquitous, but adequate sampling offsets respondent dishonesty and subjectivity. Since the questionnaire contains many questions, respondents might get tired with answering, which could lead to inaccurate answers or giving up. Moreover, the study included students from only one of the nine universities in Croatia. Therefore, the results cannot be generalised to other universities or the entire country. The study participants were recruited using a non-probability convenience sampling technique that does not provide representative results. Only students who were available and willing to participate in the study were recruited for the study. In addition, it is important to note that university students are not representative of young people in general and that attitudes may differ in other populations. Furthermore, students differ in terms of their upbringing and attitude toward dentists, which could cause a difference in their understanding of the questions and, possibly, their answers as well. This survey included only voluntary online responses, which could affect sample bias. Moreover, it was a cross-sectional study, so it is impossible to draw causal conclusions.

In dentistry, as in all other areas of medicine, patients have the same right to quality, affordable healthcare. If this type of research were conducted on the general population and at a national level, the results could be used to monitor and improve the quality of dental care.

## 5. Conclusions

We used this cross-sectional survey to determine the current level of satisfaction with dental services among students at the University of Split. The results showed that students of biomedical studies were satisfied to the same extent as students of other scientific fields in terms of interaction, technical competence, and administrative efficiency. Only minimal differences were shown in terms of pain control and the cost of dental services. Respondents were most satisfied with their dentists’ friendliness, office facilities, and the guarantee of privacy during the procedure; they were least satisfied with pain and cost. Trust, empathy, and neatness were the most important qualities that respondents sought in their choice of dentist, while the gender of the dentist was of next to no relevance. Although the overall satisfaction level of the respondents was high, there was room for improvement in several aspects of dental services, including patient–dropstaff interaction, technical competence, and administrative efficiency.

## Figures and Tables

**Table 1:** Demographic characteristics of respondents (*n* = 806).

Characteristics	Response	Frequency *n* (%)
Gender	Female	628 (77.9)
	Male	178 (22.1)
Age (years)	18–22	279 (34.6)
	23–25	441 (54.7)
	≥25	86 (10.7)
Field of study	Biomedicine and health science	456 (56.6)
	Social sciences	130 (16.1)
	Technical sciences	107 (13.3)
	Humanities	91 (11.2)
	Natural sciences	100 (12.4)
	Art	28 (3.5)
Year of study	1st year	94 (11.7)
	2nd year	125 (15.5)
	3rd year	152 (18.9)
	4th year	159 (19.7)
	5th year	207 (25.7)
	6th year	69 (8.6)
Family members employed in healthcare	No	572 (71.0)
	Yes	234 (29.0)

**Table 2:** Respondents’ attitudes regarding chosen dentist, dental practice and dental services provided (*n* = 806).

Characteristics	Response	Frequency *n* (%)
Type of dental healthcare service	Community health centers	189 (23.4)
	Private dental practice under contract with public health insurance fund–concessionaire	338 (41.9)
	Private dental practice	278 (34.1)
Satisfaction with chosen dentist and dental practice	Very dissatisfied	30 (3.7)
	Dissatisfied	41 (5.1)
	Neither satisfied nor dissatisfied	110 (13.6)
	Satisfied	295 (36.6)
	Very satisfied	330 (40.9)
Reasons for the selection of dentist and dental practice	Referral	623 (77.3)
	Advertising	7 (0.9)
	Vicinity	167 (20.7)
	Other	9 (1.1)
Would you recommend your chosen dentist?	No	98 (12.2)
	I do not know	164 (20.3)
	Yes	544 (67.5)
Have you ever changed your chosen dentist?	No	275 (34.1)
	Yes, once	261 (32.4)
	Yes, a few times	270 (33.5)
Distance of the dental facility from your residence by car (minutes)	≤5	221 (27.4)
	5–30	527 (65.4)
	30–60	36 (4.5)
	≥60	22 (2.7)
The time required to make an appointment with the chosen dentist (days)	≤7	521 (64.6)
	7–15	181 (22.5)
	16–30	79 (9.8)
	≥30	25 (3.1)
Before the procedure, the dentist asks about changes in my overall health or medicine usage	Rarely (Very Rarely)	507 (62.9)
	Never	47 (5.8)
	Always (Very Frequently)	252 (31.3)
Dental procedures last long enough and are carried out in detail	Rarely (Very Rarely)	58 (7.2)
	Never	287 (35.6)
	Always (Very Frequently)	461 (57.2)
When I come to the dental practice due to an emergency (pain, swelling, broken tooth), the dentist provides service the same day	Rarely (Very Rarely)	64 (7.9)
	Never	210 (26.1)
	Always (Very Frequently)	532 (66.0)
The information that I receive from the dentist about oral health and oral hygiene is satisfactory	Rarely (Very Rarely)	56 (6.9)
	Never	270 (33.5)
	Always (Very Frequently)	480 (59.6)

**Table 3:** Evaluation of respondents’ statements related to primary dental healthcare satisfaction.

Tested Statement	Total	Study Field		*p*
		Biomedicine and Health Science	Other Scientific Fields	
**Patient–dental staff interaction**				
Dental staff do not speak to each other while providing procedures	2.62 (1.31)	2.53 (1.25)	2.68 (1.35)	0.164
Dental staff are concentrate during procedure	4.32 (0.87)	4.31 (0.82)	4.33 (0.90)	0.299
Dental staff are friendly and approachable	4.43 (0.88)	4.47 (0.74)	4.40 (0.95)	0.724
Dental staff give explanation related to the patient’s treatment	3.84 (1.21)	3.89 (1.20)	3.80 (1.22)	0.255
Dental staff give me advice/recommendation after the procedure	4.27 (0.99)	4.32 (0.96)	4.24 (1.01)	0.253
The facial expression of dental staff during and after the procedure is friendly and with a smile	4.09 (1.08)	4.16 (1.02)	4.03 (1.12)	0.113
Dental staff do not criticize my oral health or compare it to others	4.21 (1.07)	4.22 (1.02)	4.10 (1.11)	0.715
Dental staff do not ask me personal questions during procedures	3.72 (1.31)	3.71 (1.28)	3.73 (1.34)	0.593
**Professional and technical competence of dental staff and dental surgery**				
Dental procedures are not painful	3.83 (1.05)	3.98 (0.98)	3.71 (1.09)	≤0.001
Dental staff perform detailed examination before procedures	4.24 (0.99)	4.26 (0.96)	4.23 (1.01)	0.991
I am satisfied with the quality of the services provided	4.28 (0.98)	4.29 (0.93)	4.28 (1.02)	0.532
Adequate protective equipment is used during procedures	4.72 (0.70)	4.75 (0.56)	4.69 (0.79)	0.628
Instruments, equipment and the office are clean	4.72 (0.69)	4.77 (0.55)	4.68 (0.77)	0.360
**Administrative efficiency of dental practice**				
The working hours of the dental office are satisfactory	4.49 (0.84)	4.52 (0.79)	4.45 (0.88)	0.595
I don’t have to wait long to schedule my next visit	4.14 (1.11)	4.16 (1.07)	4.12 (1.14)	0.858
I don’t wait long in the office for the scheduled treatment (the scheduled treatment time is respected)	4.14 (1.05)	4.13 (1.03)	4.14 (1.06)	0.815
Dental office is modern and technically well-equipped	4.27 (0.96)	4.31 (085)	4.24 (1.04)	0.848
The waiting room is comfortable and pleasant	4.12 (1.07)	4.13 (1.00)	4.12 (1.13)	0.510
Privacy during the treatment is ensured	4.50 (0.82)	4.77 (0.74)	4.46 (0.88)	0.157
The prices of dental services are high	2.91 (1.28)	2.77 (1.20)	3.01 (1.32)	0.007

Data are presented as median value (standard deviation). Statistical significance was tested with Mann-Whitney test. Statistical significance is set to *p* < 0.05.

**Table 4:** Correlation between respondents’ attitudes related to satisfaction with the dental service provided in primary healthcare and their sociodemographic data.

Tested Statement	Gender	Age	Study Field	Year of Study	Family Members Working in Healthcare
	*r* (*p*)	*r* (*p*)	*r* (*p*)	*r* (*p*)	*r* (*p*)
**Patient-dental staff interaction**					
Dental staff do not speak to each other during procedures	0.025 (0.447)	−0.036 (0.305)	0.049 (0.164)	−0.004 (0.906)	−0.046 (0.197)
Dental staff are concentrate during procedure	0.030 (0.389)	0.025 (0.480)	0.037 (0.299)	−0.004 (0.912)	0.007 (0.834)
Dental staff are friendly and approachable	−0.005 (0.882)	0.004 (0.905)	0.012 (0.724)	0.002 (0.964)	0.073 (0.037)
Dental staff give explanation related to the patient’s treatment	−0.028 (0.427)	0.009 (0.808)	−0.040 (0.256)	−0.020 (0.557)	0.040 (0.258)
Dental staff give me advice/recommendation after the procedure	−0.010 (0.784)	−0.043 (0.224)	−0.040 (0.254)	−0.034 (0.331)	0.075 (0.034)
The facial expression of the dental staff during and after the procedure is friendly and with a smile	−0.033 (0.348)	0.020 (0.569)	−0.056 (0.113)	0.058 (0.101)	0.035 (0.319)
Dental staff do not criticize my oral health or compare it to others	−0.089 (0.011)	0.005 (0.890)	0.013 (0.716)	0.023 (0.518)	0.022 (0.540)
Dental staff do not ask me personal questions during procedures	−0.023 (0.506)	0.072 (0.041)	0.019 (0.593)	0.088 (0.012)	−0.023 (0.507)
**Professional and technical competence of dental staff and dental office**					
Dental procedures are not painful	−0.062 (0.077)	0.012 (0.743)	−0.121 (<0.001)	0.039 (0.266)	0.051 (0.151)
Dental staff perform a detailed examination before procedures	0.023 (0.510)	−0.006 (0.869)	−0.004 (0.911)	−0.003 (0.925)	0.016 (0.648)
I am satisfied with the quality of the services provided	0.008 (0.815)	−0.035 (0.315)	0.022 (0.533)	−0.038 (0.286)	0.024 (0.499)
Adequate protective equipment is used during procedures	−0.036 (0.306)	−0.022 (0.530)	0.017 (0.628)	−0.050 (0.152)	−0.007 (0.848)
Instruments, equipment and the office are clean	−0.050 (0.157)	−0.043 (0.222)	−0.032 (0.361)	−0.049 (0.162)	0.055 (0.121)
**Administrative efficiency of dental practice**					
The working hours of the dental office are satisfactory	−0.044 (0.209)	−0.052 (0.137)	−0.019 (0.596)	−0.014 (0.688)	0.006 (0.871)
I don’t have to wait long to schedule my next visit	0.047 (0.183)	−0.025 (0.483)	−0.006 (0.858)	0.027 (0.450)	0.007 (0.853)
I don’t wait long in the office for the scheduled treatment (the scheduled treatment time is respected)	0.030 (0.390)	−0.020 (0.568)	0.008 (0.815)	−0.022 (0.525)	0.028 (0.429)
Dental office is modern and technically well-equipped	−0.022 (0.529)	−0.122 (≤0.001)	0.007 (0.847)	−0.100 (0.004)	0.045 (0.202)
The waiting room is comfortable and pleasant	−0.034 (0.331)	−0.104 (0.003)	0.023 (0.510)	−0.064 (0.070)	0.068 (0.055)
Privacy during the treatment is ensured	0.053 (0.134)	−0.023 (0.513)	−0.050 (0.157)	−0.010 (0.770)	0.044 (0.208)
The prices of dental services are high	0.063 (0.072	0.065 (0.067)	0.095 (0.007)	0.039 (0.267)	0.017 (0.624)

Spearman’s correlation. Statistical significance was set at *p* < 0.05.

**Table 5:** Evaluation of the most important reason for choosing a dentist.

Characteristics	Total	Study Field		*p*
		Biomedicine and Health Science	Other Scientific Fields	
Quality and efficiency of work	4.87 (0.43)	4.88 (0.41)	4.87 (0.44)	0.896
Experience	4.28 (0.88)	4.23 (0.85)	4.32 (0.89)	0.031
Reputation	4.00 (1.00)	3.94 (0.99)	4.05 (1.00)	0.068
Price service	3.78 (1.01)	3.67 (0.93)	3.86 (1.06)	≤0.001
Technical equipment of the dental office	4.06 (0.84)	4.03 (0.79)	4.07 (0.88)	0.226
Neatness and cleanliness of the dental office and the dentist	4.74 (0.55)	4.77 (0.46)	4.71 (0.60)	0.542
Trust and empathy	4.58 (0.71)	4.62 (0.62)	4.56 (0.77)	0.831
Communication skills	4.30 (0.85)	4.40 (0.74)	4.22 (0.92)	0.017
Approachability and friendly attitude	4.51 (0.75)	4.58 (0.64)	4.46 (0.81)	0.097
Dental office location	3.47 (1.13)	3.38 (1.09)	3.54 (1.15)	0.034
Waiting time	3.90 (0.93)	3.83 (0.88)	3.95 (0.96)	0.029
Office working hours	3.47 (1.05)	3.45 (1.02)	3.48 (1.07)	0.446
Professional competence of dentist	4.19 (0.86)	4.23 (0.85)	4.34 (0.86)	0.033
Dentist gender	1.43 (0.91)	1.13 (0.77)	1.51 (1.01)	0.007

Data are presented as median value (standard deviation). Statistical significance was tested with Mann-Whitney test. Statistical significance is set to *p* < 0.05.

**Table 6:** Correlation between in the most important reason for choosing the dentist and the respondent’s sociodemographic data.

Characteristics	Gender	Age	Study Field	Year of Study	Family Members Working in Healthcare
	*r* (*p*)	*r* (*p*)	*r* (*p*)	*r* (*p*)	*r* (*p*)
Quality and efficiency of work	−0.081 (0.218)	0.011 (0.862)	−0.077 (0.242)	0.024 (0.713)	0.040 (0.252)
Experience	−0.012 (0.724)	0.010 (0.780)	0.076 (0.031)	−0.018 (0.605)	−0.008 (0.823)
Reputation	0.031 (0.374)	−0.004 (0.901)	0.064 (0.068)	0.005 (0.895)	0.013 (0.708)
Price service	−0.023 (0.504)	0.043 (0.224)	0.121 (≤0.001)	0.055 (0.119)	−0.090 (0.011)
Technical equipment of the dental office	−0.060 (0.089)	−0.058 (0.101)	0.043 (0.227)	−0.039 (0.265)	−0.052 (0.139)
Neatness and cleanliness of the dental office and the dentist	−0.152 (≤0.001)	−0.065 (0.065)	−0.021 (0.542)	−0.076 (0.032)	0.021 (0.545)
Trust and empathy	−0.167 (≤0.001)	−0.007 (0.833)	−0.008 (0.831)	−0.036 (0.303)	0.083 (0.018)
Communication skills	−0.208 (≤0.001)	−0.005 (0.897)	−0.084 (0.017)	−0.003 (0.943)	0.080 (0.023)
Approachability and friendly attitude	−0.209 (≤0.001)	0.010 (0.768)	−0.059 (0.097)	0.008 (0.816)	0.126 (≤0.001)
Dental office location	−0.039 (0.263)	0.013 (0.709)	0.075 (0.034)	−0.012 (0.736)	−0.030 (0.392)
Waiting time	−0.053 (0.134)	−0.010 (0.784)	0.077 (0.029)	0.014 (0.693)	−0.025 (0.481)
Office working hours	−0.035 (0.317)	0.020 (0.570)	0.026 (0.467)	0.039 (0.265)	0.020 (0.571)
Professional competence of dentist	−0.060 (0.086	−0.059 (0.094)	0.075 (0.033)	−0.021 (0.559)	−0.002 (0.963)
Dentist gender	−0.006 (0.870	0.024 (0.492)	0.095 (0.007)	−0.008 (0.822)	−0.061 (0.085)

Spearman’s correlation. Statistical significance was set at *p* < 0.05.

## Data Availability

The data will be shared by the authors of this research paper upon request.
